# Financial strain, neighborhood cohesion, and health-related quality of life among rural and urban Spanish-speaking Latina breast cancer survivors

**DOI:** 10.1007/s11764-023-01369-2

**Published:** 2023-03-29

**Authors:** Jasmine Santoyo-Olsson, Anita L. Stewart, Anna María Nápoles

**Affiliations:** 1 University of California San Francisco, Division of Internal Medicine, 490 Illinois Street, 9th floor, Box 0856, San Francisco, CA 94158 USA; 2 University of California Berkeley, School of Public Health, 2121 Berkeley Way, Room 5302, Berkeley, CA 94720 USA; 3 University of California San Francisco, Institute for Health & Aging, Center for Aging in Diverse Communities, 490 Illinois Street, 12th floor, Box 0646, San Francisco, CA 94158 USA; 4 Division of Intramural Research, National Institute On Minority Health and Health Disparities, National Institutes of Health, 9000 Rockville Pike, Building 3, Floor 5, Room E08, Bethesda, MD 20892 USA

**Keywords:** Quality of life, Breast neoplasm, Financial strain, Neighborhood cohesion, Rural, Urban

## Abstract

**Purpose:**

Among Latina breast cancer survivors, explore associations between rural/urban residence and health-related quality of life (HRQL), and whether associations are moderated by financial strain and low neighborhood cohesion.

**Methods:**

We combined baseline data from two randomized controlled trials of a stress management intervention conducted among 151 urban and 153 rural dwelling Latinas with nonmetastatic breast cancer. Generalized linear models estimated associations between rural/urban status and HRQL (overall, emotional, social-family, physical, and functional well-being), and we examined moderation effects of financial strain and low neighborhood cohesion, controlling for age, marital status, and breast cancer characteristics.

**Results:**

Rural women reported better emotional (β = 1.85; 95% CI = 0.37, 3.33), functional (β = 2.23; 95% CI = 0.69, 3.77), and overall (β = 5.68; 95% CI = 1.12, 10.25) well-being than urban women, regardless of degree of financial strain or neighborhood cohesion; moderation effects were not statistically significant. Financial strain was inversely associated with emotional (β = -2.34; 95% CI = 3.63, -1.05), physical (β = -2.56; 95% CI = -4.12, -1.01), functional (β = -1.61; 95% CI = -2.96, -0.26), and overall (β = -6.67; 95% CI = -10.96, -2.98) well-being. Low neighborhood cohesion was inversely associated with emotional (β = -1.27; 95% CI = -2.50, -0.04), social-family (β = -1.72; 95% CI = -3.02, -0.42), functional (β = -1.63; 95% CI = -2.92, -0.34), and overall (β = -5.95; 95% CI = 9.76, -2.14) well-being.

**Conclusions:**

Rural Latina breast cancer survivors reported better emotional, functional and overall well-being than their urban counterparts. Greater financial strain and less neighborhood cohesion were associated with worse HRQL on most domains regardless of rural/urban context.

**Implications for Cancer Survivors:**

Interventions that focus on increasing perceived neighborhood cohesion and reducing or better managing financial strain, could help improve Latina cancer survivors’ well-being.

## Introduction

Breast cancer is the most commonly diagnosed cancer among Latina women [1]. Despite improvements in breast cancer detection practices and treatments, Latinas are more likely to be diagnosed with more advanced stage breast cancer and less likely to receive appropriate and timely breast cancer treatment than White women [2]. Also, compared to other racial/ethnic groups with cancer, Latina breast cancer survivors report worse health-related quality-of-life (HRQL) [3, 4]. Latina women with breast cancer experience poorer physical health, and higher symptom burden and social disruption compared with cancer survivors of other racial/ethnic groups [3, 4], factors that contribute to worse HRQL. These psychosocial inequities among Latina cancer survivors are often attributed to upstream factors such as job disruption, financial strain, lower income and education, worse access to healthcare and cancer treatments, and limited English proficiency [3, 4]. Spanish-speaking Latina women with breast cancer may experience distress associated with lack of English fluency, compounding the difficulties they experience when accessing and utilizing healthcare services [5].

An especially important factor affecting the HRQL of Latinas with breast cancer is financial strain. Financial strain is a growing problem for people with cancer and their families, regardless of cancer type [6, 7]. Increased financial strain due to cancer can lead to worse HRQL [8, 9]. Moreover, financial strain due to cancer and its treatment has adverse effects on household income [8], medication adherence [9], maintaining housing and preventing eviction or foreclosure [10], transportation [10], and family relationships [11]. Compared with White cancer survivors, Latino cancer survivors more often report loss of employment-related income and health insurance [12] that can affect adversely Latino cancer survivors’ HRQL [4]. While financial strain due to cancer is not unique to Latina cancer survivors, it has greater impact because median personal income is on average lower for Latinos compared to other racial/ethnic groups [13].

A person’s geographic location may also affect their cancer specific HRQL. Compared to those from urban areas, rural residents have access to fewer health care workers per capita, lack of specialty cancer care, fewer social workers and mental health providers per capita, and longer travel times to providers and/or diagnostic and treatment facilities [14–16]. Less is known about the influence of geographic location (rural, urban) on the HRQL of Latina women with breast cancer [17, 18]; most studies have focused on those living in urban locations [3, 19] or urban–rural White comparisons [20, 21]. Among breast cancer survivors in general, greater rurality of an area is inversely associated with overall HRQL and social functioning, and positively associated with financial strain and breast cancer symptoms [20, 21]. Shortages of bilingual/bicultural providers and interpreter services in rural areas limit access to cancer psychosocial services [22]. Thus, there is a need to examine the association of rural/urban geographic location on HRQL among Latinas with breast cancer.

Neighborhood cohesion is a residential area characteristic that could affect HRQL among cancer survivors. Neighborhood cohesion is the perceived degree of connection among neighbors, and people’s willingness to intervene for the common good [23]. Neighborhood cohesion has been studied in the context of access to cancer screening [24], but less is known about its effects on the HRQL of breast cancer survivors. Neighborhood cohesion may be especially relevant to Latinos because they tend to cluster in close geographic spaces with high concentrations of people belonging to the same ethnic group. A nationally representative survey of Latino individuals found that neighborhood cohesion was positively related to self-rated physical and mental health [25]. Greater neighborhood stress was associated with poorer self-reported health in a study examining neighborhood context and health outcomes among urban Black women and English- and Spanish-speaking Latina women with breast cancer [26]. However, the effects of neighborhood cohesion on HRQL among Latinas with breast cancer and whether these effects differ by rural versus urban setting have received scarce attention.

In this paper, we used baseline data from two randomized controlled trials (RCTs) that tested the effects of a stress management program on HRQL, one conducted in rural communities and the other in urban communities. We cross-sectionally examined: 1) differences in HRQL among rural and urban Latina breast cancer survivors; 2) associations of financial strain and low neighborhood cohesion with HRQL overall and by rural/urban location; and 3) whether financial strain or low neighborhood cohesion moderate the rural/urban associations. We hypothesize that rural (vs. urban) residence will be associated with worse HRQL, and that this association will be stronger among Latina breast cancer survivors reporting higher levels of financial strain and lower levels of neighborhood cohesion.

## Methods

### Data sources

This study is a cross-sectional secondary data analysis of aggregated data from two 6-month RCTs conducted among Latina, non-metastatic breast cancer survivors, the *Nuevo Amanecer* urban [27] and rural [28] studies. Both studies assessed the effectiveness of a culturally adapted stress management intervention on breast cancer-specific quality of life and psychological distress. The first study was conducted among 151 Latinas living in five urban [27] California counties and the second occurred among 153 Latinas living in three rural California counties [28]. The counties that were designated as rural areas are characterized by an economy that is heavily based on agriculture and with large Latino immigrant populations – Imperial County (85% Latino), Tulare County (66% Latino), and cities of Watsonville (81% Latino) and Salinas (82% Latino) [29]. The California counties that were classified as urban in our study were Alameda County (22% Latino), Contra Costa County (26% Latino), San Francisco County (15% Latino), San Mateo County (24% Latino), and Santa Clara County (25% Latino), and consist of major metropolitan areas [29].

Both of the original RCT studies were approved by the University of California San Francisco Institutional Review Board (#10–03030 and #16–18737), but the secondary data analyses presented here were considered non-human subjects research because we used de-identified data.

### Sample

Both studies enrolled primarily monolingual (85%) Spanish-speaking Latinas with non-metastatic breast cancer utilizing identical recruitment and data collection methods, although inclusion criteria differed slightly between the studies. The first study occurring in 2011–2014 among women living in the five urban counties and was restricted to those who had been diagnosed in the past year. In the second study conducted in 2016–2018 among women living in the three rural communities, eligibility was not restricted based on time since diagnosis, i.e., open to women regardless of time since diagnosis. In both studies, community-based organizations with networks to oncology clinics and hospitals were the primary sources of recruitment. Community-based recruiters who worked with community organizations serving Latinos with cancer contacted women in person or by telephone, verified their eligibility, and conducted the baseline survey in person prior to randomization. Additional details on recruitment of the samples and original studies are available elsewhere [27, 28].

### Measures

Survey measures were self-reported and assessed HRQL, financial strain, neighborhood cohesion, and sociodemographic and breast cancer characteristics; measures were identical across both studies.

#### Health-related quality of life: dependent variables

The Functional Assessment of Cancer Therapy-General (FACT-G) was used to measure health-related quality of life [30] and has been translated and validated in Spanish [31]. The FACT-G consists of four subscale scores pertaining to emotional, social-family, physical, and functional well-being domains). A total overall score consists of the sum of the subscale scores. Subscales were scored by summing items after reversing some items; higher scores indicate greater well-being. Possible score ranges are emotional well-being, 0–24 (internal consistency reliability = 0.77); social-family well-being, 0–28 (internal consistency reliability = 0.76); physical well-being, 0–28 (internal consistency reliability = 0.87); and functional well-being, 0–28 (internal consistency reliability = 0.84). The FACT-G total score has a possible range of 0–108 (internal consistency reliability = 0.72).

#### Rural/urban residence: independent variable

Rural/urban residence was coded dichotomously with urban location as the reference group. Rural and urban county-level classifications were consistent with their designation by the Medical Service Study Areas (MSSAs) taxonomy developed by the California Office of Statewide Health Planning and Development, which are used to identify medically underserved areas [32].

#### Hypothesized moderators: financial strain and neighborhood cohesion

Financial strain was assessed using a summary variable derived from three items that asked women if in the past month, they ever did not have enough money to pay for: their monthly bills, medical visits for breast cancer, and prescription medicines for breast cancer (0 = no; 1 = yes). The total score was the sum of “yes” responses (range = 0–3). Based on frequency distributions, we dichotomized the total score as no (reference group) versus any financial strain.

Neighborhood cohesion was assessed using a summary variable derived from three items that asked respondents the extent to which they agree/disagree with statements describing the neighborhood in which they live: people in this neighborhood can be trusted, are willing to help, and this is a close-knit neighborhood [33]. Response options were 1 = strongly disagree, 2 = disagree, 3 = neutral (neither agree nor disagree), 4 = agree, and 5 = strongly agree. A summary score was calculated as the average of non-missing items. Scores ranged from 1–5, and higher scores indicate greater cohesion (internal consistency reliability = 0.85). For multivariate analyses, based on frequency distributions, we dichotomized the total score at the mean (mean = 3.42, SD = 1.06) as high cohesion =  ≥ 3.42 (reference group) and low cohesion = 1 to < 3.42.

#### Sociodemographic characteristics

Sociodemographic variables of age in years at baseline (continuous), marital status (separated/divorced/widowed/never married versus married/living with a partner), English proficiency (not at all/poorly/fairly well versus well/very well), self-identified ethnicity (Mexican, Central American, South American, Other Latina), employment status (unemployed/homemaker/caregiver/retired/disabled versus employed full or part time), years living in the U.S.(continuous), and educational attainment (less than high school, high school graduate, or more than high school graduate) were included.

#### Breast cancer characteristics

We assessed several breast cancer-related characteristics: time in months (continuous) since breast cancer diagnosis; breast cancer stage (0, I, II or III; women with metastatic cancer were excluded from the original RCT studies); type of surgery received for breast cancer (lumpectomy, mastectomy, or none); and breast cancer treatment (both chemotherapy and radiation, radiation only, chemotherapy only, or no treatment).

### Data analysis

Descriptive statistics were used to characterize the sample overall and by rural/urban location. To examine differences in HRQL, financial strain, and low neighborhood cohesion by rural/urban location, T-tests were used. To test for independent associations of rural/urban location, financial strain, and low neighborhood cohesion with each of the HRQL outcomes, separate generalized linear models for each of the outcomes of emotional well-being, social-family well-being, physical well-being, functional well-being, and overall FACT-G were estimated, controlling for age, marital status, and breast cancer characteristics (stage, type of surgery, treatment, and length of time since diagnosis). Only variables that were significantly associated with rural/urban location in bivariate analyses or supported by the literature were included as covariates. To test whether associations between rural/urban location and each HRQL outcomes vary depending on presence of financial strain or low neighborhood cohesion (moderation effects), models with interaction terms (financial strain x rural/urban and low neighborhood cohesion x rural/urban) were conducted first. Interaction terms that were not statistically significant were dropped from the final models. All analyses were performed using SAS (version 9.4).

## Results

On average, women were 52.7 (SD = 10.9) years of age (Table 1). The majority of women were married or living with a partner (60%), non-English proficient (85%), most were Mexican (82%), were not employed (82%), and most had less than high school education (68%). On average women had lived in the U.S. for 23.3 years (SD = 13.1). Rural women tended to be older (mean age 54.8 vs. 50.5 years; *p* < 0.01), more likely to be married (66% vs. 53%; *p* < 0.05) and had lived longer in the U.S. (mean years 26.7 vs. 20.3; *p* < 0.0001).

**Table 1:** Demographic and breast cancer sample characteristics, health-related quality of life, financial strain and neighborhood cohesion, overall and by rural and urban geographic location, *Nuevo Amanecer* studies

	Total *N* = 304	Rural *n* = 153	Urban *n* = 151	p-value
Socio-Demographic Characteristics				
Age at baseline, mean (SD)	52.7 (10.9)	54.8 (10.5)	50.5 (10.9)	< .001
Marital status, *n* (%)				
Separated/divorced, widowed, or single Married or living with a partner	123 (40) 181 (60)	52 (34) 101 (66)	71 (47) 80 (53)	< .05
English proficiency, *n* (%)				
Not at all/poorly/fairly well Well/very well Missing data	257 (85) 46 (15) 1 (0)	124 (81) 28 (18) 1 (1)	133 (88) 18 (12) 0 (0)	0.18
Ethnicity, *n* (%)				
Mexican Central American South American Other Latina	250 (82) 36 (12) 14 (5) 4 (2)	149 (97) 1 (1) 0 (0) 3 (2)	101 (67) 35 (23) 14 (9) 1 (1)	< .0001
Employment status, *n* (%)				
Unemployed, caregiver, retired, or disabled Employed full or part time Missing data	248 (82) 54 (18) 2 (1)	125 (82) 28 (18) 0 (0)	123 (81) 26 (17) 2 (1)	0.35
Years living in the U.S., mean (SD)	23.3 (13.1)	26.7 (13.9)	20.3 (11.5)	< .0001
Educational attainment, *n* (%)				
Less than high school High school graduate More than high school graduate Missing	206 (68) 44 (14) 53 (17) 1 (0)	106 (69) 17 (11) 29 (19) 1 (1)	100 (66) 27 (18) 24 (16) 0 (0)	0.27
Breast Cancer-Related Characteristics				
Months since breast cancer diagnosis, mean (SD)	19.8 (31.7)	35.5 (38.7)	3.82 (2.7)	< .0001
Breast cancer stage, *n* (%)				
0 I II III Missing data	48 (16) 68 (22) 112 (37) 58 (19) 18 (6)	8 (5) 45 (29) 55 (36) 27 (18) 18 (12)	40 (26) 23 (15) 57 (38) 31 (21) 0 (0)	< .0001
Type of surgery received, *n* (%)				
Lumpectomy Mastectomy None/Missing data^a^	160 (53) 139 (46) 5 (2)	76 (50) 72 (47) 5 (3)	84 (56) 67 (44) 0 (0)	0.06
Breast cancer treatment received, *n* (%)				
Both chemotherapy and radiation Radiation only Chemotherapy only No treatment Missing data	151 (50) 70 (23) 47 (15) 34 (11) 2 (1)	91 (60) 28 (18) 22 (14) 10 (7) 2 (1)	60 (40) 42 (28) 25 (17) 24 (16) 0 (0)	< .01
Outcomes and Independent Variables				
Emotional well-being, mean (SD)	16.1 (5.2)	17.3 (4.6)	14.9 (5.4)	< .0001
Social-family well-being, mean (SD)	17.8 (5.6)	18.2 (5.2)	17.3 (5.9)	0.20
Physical well-being, mean (SD)	19.5 (6.3)	20.4 (6.3)	18.6 (6.2)	< .05
Functional well-being, mean (SD)	16.7 (5.5)	18.2 (5.1)	15.2 (5.5)	< .0001
Total FACT-G, mean (SD)	70.1 (16.6)	74.0 (15.9)	66.1 (16.3)	< .0001
Financial strain status, *n* (%)				
None Financially strained Missing data	115 (38) 186 (61) 3 (1)	79 (52) 74 (48) 0 (0)	36 (24) 112 (74) 3 (2)	< .0001
Neighborhood Cohesion, *n* (%)				< .01
Low cohesion High cohesion Missing	159 (52) 144 (47) 1 (1)	65 (42) 87 (57) 1 (1)	94 (62) 57 (38) 0 (0)	

SD = standard deviation

^a^One person reported having no surgery

### Overall health-related quality of life, financial strain, and neighborhood cohesion

In the total sample, the full range was observed for the emotional and physical well-being subscales, and close to the full range for social-family well-being (range 1–28) and functional well-being (range 2–28). The total FACT-G range was 23–108. The mean scores for HRQL subscales were: emotional well-being, 16.1 (SD = 5.2); social-family well-being, 17.8 (SD = 5.6); physical well-being, 19.5 (SD = 6.3); and functional well-being, 16.7 (SD = 5.5). The mean score for the total FACT-G score was 70.1 (SD = 16.6).

Overall, over half (61%) of the women reported experiencing financial strain and low neighborhood cohesion (52%).

### Rural/Urban differences in HRQL, financial strain, and neighborhood cohesion

In bivariate analyses, women in rural areas reported significantly better emotional well-being (*p* < 0.0001), physical well-being (*p* < 0.05), functional well-being (*p* < 0.0001), and total FACT-G (*p* < 0.0001) scores than those in urban locations (Table 1). Women in rural areas were less likely to report financial strain (48% vs 74%; *p* < 0.0001) and more likely to report high neighborhood cohesion (57% vs. 38%; *p* < 0.01) than women in urban areas.

### Multivariate models predicting HRQL

There was no effect modification by financial strain or low neighborhood cohesion of the associations between rural/urban residence and any of the HRQL outcomes (interaction terms were non-significant; p values > 0.38). Therefore, these interaction terms were dropped from the final models.

In the final model (Table 2) for emotional well-being, living in a rural area (β = 1.85; 95% confidence interval (CI) = 0.37, 3.33) was positively associated, and financial strain (β = -2.34; 95% CI = 3.63, -1.05) and neighborhood cohesion (β = -1.27; 95% CI = -2.50, -0.04) were inversely associated with emotional well-being.

**Table 2:** Multivariate associations of rural/urban location, financial strain, and low neighborhood cohesion with health-related quality of life by domain, *Nuevo Amanecer* studies

	Multivariate Model		
	β	p	95% CI
Emotional well-being	R^2^ = 0.147, F(13, 265) = 3.51, *p* < .0001		
Rural (ref. urban)	**1.85**	** < .05**	**(0.37, 3.33)**
Financial strain (ref. none)	**-2.34**	** < .001**	**(-3.63, -1.05)**
Low neighborhood cohesion (ref. high)	**-1.27**	** < .05**	**(-2.50, -0.04)**
Social-family well-being	R^2^ = 0.181, F(13, 265) = 4.51, *p* < .0001		
Rural (ref. urban)	0.61	0.44	(-0.95, 2.17)
Financial strain (ref. none)	-0.45	0.52	(-1.82, 0.91)
Low neighborhood cohesion (ref. high)	**-1.72**	** < .01**	**(-3.02, -0.42)**
Physical well-being	R^2^ = 0.166, F(13, 265) = 4.07,* p* < .0001		
Rural (ref. urban)	1.00	0.27	(-0.77, 2.77)
Financial strain (ref. none)	**-2.56**	** < .01**	**(-4.12, -1.01)**
Low neighborhood cohesion (ref. high)	-1.33	0.08	(-2.81, 0.15)
Functional well-being	R^2^ = 0.168, F(13, 265) = 4.13, *p* < .0001		
Rural (ref. urban)	**2.23**	** < .01**	**(0.69, 3.77)**
Financial strain (ref. none)	**-1.61**	** < .05**	**(-2.96, -0.26)**
Low neighborhood cohesion (ref. high)	**-1.63**	** < .05**	**(-2.92, -0.34)**
FACT-G	R^2^ = 0.205, F(13, 265) = 5.25, *p* < .0001		
Rural (ref. urban)	**5.68**	** < .05**	**(1.12, 10.25)**
Financial strain (ref. none)	**-6.97**	** < .001**	**(-10.96, -2.98)**
Low neighborhood cohesion (ref. high)	**-5.95**	** < .01**	**(-9.76, -2.14)**

Ref. = reference group

^*^Controlled for age, marital status, and breast cancer characteristics (stage, type of surgery, treatment, and length of time since diagnosis)

The bold indicates the independent variables that were significant for each model per outcome of interest

Only low neighborhood cohesion (β = -1.72; 95% CI = -3.02, -0.42) was inversely associated with social-family well-being. Neither rural location nor financial strain was significantly associated with social-family well-being.

Only financial strain (β = -2.56; 95% CI = -4.12, -1.01) was inversely associated with physical well-being. Neither rural location nor low neighborhood cohesion was significantly associated with physical well-being.

For functional well-being, living in a rural area (β = 2.23; 95% CI = 0.69, 3.77) was positively associated, and financial strain (β = -1.61; 95% CI = -2.96, -0.26) and low neighborhood cohesion (β = -1.63; 95% CI = -2.92, -0.34) were inversely associated with functional well-being.

For the summary FACT-G measure, living in a rural area (β = 5.68; 95% CI = 1.12, 10.25) was positively associated, and financial strain (β = -6.67; 95% CI = -10.96, -2.98) and low neighborhood cohesion (β = -5.95; 95% CI = -9.76, -2.14) were inversely associated with FACT-G.

## Discussion

In this study, we aimed to assess associations between rural/urban residence and several dimensions of breast cancer related HRQL, and whether these associations are moderated by financial strain and neighborhood cohesion among Latina breast cancer survivors. We hypothesized that rural (vs. urban) residence would be associated with worse HRQL, and that this association would be stronger among Latina breast cancer survivors reporting higher levels of financial strain and lower levels of neighborhood cohesion. However, in our study, rural women reported better emotional, functional, and overall HRQL than urban women, regardless of degree of financial strain or neighborhood cohesion. Financial strain was inversely associated with emotional, physical, functional, and overall (all except for social-family) well-being. Low neighborhood cohesion was inversely associated with emotional, social-family, functional, and overall (all except for physical) well-being.

Studies comparing rural and urban cancer survivors on HRQL have had inconsistent findings. In fact, two systematic reviews of studies focusing on predominantly White samples with various cancer types reached opposite conclusions [18, 34]. One review found that five studies reported poorer psychosocial outcomes (social and emotional functioning) among urban cancer survivors, while three studies reported poorer outcomes (physical functioning, role functioning and mental health issues) among cancer survivors living in rural areas, leading them to conclude that neither demonstrated a clear advantage [34]. The other systematic review of HRQL among urban and rural cancer survivors found that the majority of studies reported worse psychosocial outcomes among rural survivors compared to their urban dwelling counterparts [18]. In addition to the lack of ethnic diversity, studies included English-speaking people, which limits generalizability to people with limited English proficiency.

Our study found better HRQL among rural Latina breast cancer survivors, which ran counter to our hypotheses; we predicted that rural dwelling survivors would have worse HRQL because of the resource constraints faced by rural residents and health care systems. A possible explanation for the better HRQL observed in our study among the rural women could be due to the positive effects of ethnic enclaves, which allow individuals and neighborhoods to maintain essential functions, such as economic growth and social networks, without requiring English ability to navigate those functions [35]. Between 2000–2017, rural areas of California experienced significant growth of their Latino populations [36], which may have increased the penetrance of ethnic enclaves. More affordable housing in rural areas has been a driving force for outmigration from the San Francisco Bay Area where our urban study sites were located to the rural areas [37, 38]. In our study, rural women also reported less financial strain than urban women, which could help explain their better HRQL. Furthermore, in our study, more rural women were married or living with a partner than urban women. Therefore, better HRQL among the rural women could be due in part to the greater availability of support from a partner. Importantly, our findings need replication by other studies of Latino cancer survivors using consistent measures of HRQL and related outcomes.

Rural populations are often characterized as more stoic and self-resilient, relying little on formal support and using their own mental resources and strategies to cope with challenges [39]. Resources that may protect HRQL among rural cancer survivors, but not urban cancer survivors include coping strategies to cope with cancer such as “active coping,” “positive reinterpretation,” [40] or maintaining a “positive attitude” [41]. Lastly, evidence also suggest that longer term cancer survivors report higher HRQL [42]. In our sample, rural women were further from their time of diagnosis than urban women.

With few exceptions, the important influences of financial strain and low neighborhood cohesion on HRQL were supported in our study. Financial strain and low neighborhood cohesion affected every domain of HRQL in the expected directions (inversely for both financial strain and low neighborhood cohesion), except for social well-being in the case of financial strain and physical well-being for low neighborhood cohesion. Importantly, both of these factors were associated with HRQL regardless of whether women resided in rural or urban settings.

Financial strain is a significant problem for people with cancer regardless of insurance type, insurance literacy, or financial literacy [6, 7, 43]. Increased financial strain due to cancer can lead to worse HRQL [8, 9]. System level efforts by health plans, hospitals, and federal government, as well as financial navigation programs are needed to better address financial strain among vulnerable cancer patients [43]. Our findings that low neighborhood cohesion was associated with poorer HRQL is consistent with past studies. For example, greater neighborhood stress was associated with poorer self-reported health in a study examining neighborhood context and health outcomes among urban Black women and English- and Spanish-speaking Latina women with breast cancer [26]. Further, neighborhood characteristics such as neighborhood socioeconomic status racial/ethnic composition, population density, and degree of urbanicity have been shown to affect HRQL among various cancer survivors [44–46].

Our results have implications for addressing cancer health inequities among vulnerable Latina breast cancer survivors. Efforts to understand HRQL inequities experienced by Latina women with breast cancer need to consider the important roles of financial inequities in increasing risk and the potential protective effects of neighborhood cohesion. Interventions to improve HRQOL among cancer survivors should consider the neighborhood context as the degree of financial strain and neighborhood cohesion vary across communities. Furthermore, embedding cancer support programs in community settings could be especially critical among rural Latina women experiencing additional challenges accessing health care and necessary support services due to long travel times and limited number of health care facilities or mental health providers [14], such as National Cancer Institute (NCI)-designated cancer centers [47]. Psychosocial cancer support programs that do not consider the role of socioeconomic factors, neighborhood resources that can be harnessed, and geographic location may allocate resources and implement interventions that unintentionally reinforce or increase, psychosocial cancer disparities among Latina women.

## Limitations

Although this study makes several important contributions to psychosocial research on rural/urban location, financial strain and neighborhood cohesion, some limitations exist. First, this study uses a cross-sectional design which limits our ability to make causal inferences. Another limitation is the self-report nature of financial strain and neighborhood cohesion; we lacked objective measures of these variables. Furthermore, the results may not be generalizable to other groups of Latina women since our samples were limited largely to Spanish-speaking Mexican immigrant Latina women. In addition, rural and urban women in our sample were recruited from community-based organizations with connections to hospitals and oncology clinics. Thus, women in both samples may have been better connected to cancer survivorship resources than the general Latina breast cancer survivor population in California. Future research could collect data on systemic variables, such as neighborhood socioeconomic and built environment variables to better understand how these factors that influence on HRQL vary by geographic location [48].

## Conclusions

Rural Latina women with breast cancer tended to report better emotional, functional and overall well-being than their urban counterparts. Experiencing greater financial strain and less neighborhood cohesion were associated with worse HRQL on most domains regardless of rural or urban context. Thus, testing psychosocial cancer support interventions that foster a sense of neighborhood cohesion and address economic and geographic contexts among Latina women with breast cancer may help ameliorate psychosocial health inequities among this population of vulnerable cancer survivors in both rural and urban areas.

## Data Availability

The datasets analysed during the current study are available from the corresponding author on reasonable request.
